# Supplementary material for: Content-rich biological network constructed by mining PubMed abstracts
Source: BMC Bioinformatics. 2004 Oct 8;5:147. doi: 10.1186/1471-2105-5-147 (PMC528731; doi:10.1186/1471-2105-5-147)
Supplement: Additional File 2 — The original results of the above study (non-essential files are deleted to keep the file size under the limit set by BMC bioinformatics). [file 1471-2105-5-147-S2.bz2 › chilibotAdditionalFile2/dip05/636ID11183778E11023/html/GTF2F1_GTF2B.html]

 


 **GTF2F1** and **GTF2B** 
  
Found 80 abstracts in PubMed, retrieved 05.  
 

 What does Google say? 
 PDF only 
| .edu only 

---

**Interactive relationship** (e.g. stimulation, inhibition, etc)

**Inhibitory relationship**- The phosphatase activity of FCP1 is strongly enhanced by the carboxyl terminal domain of RAP74  [ **GTF2F1** ]  cterRAP74, residues 436 517, and this stimulatory effect of TFIIF  [ **GTF2F1** ]  can be blocked by TFIIB  [ **GTF2B** ] .  Ref: 12578358 Biochemistry< MedlineTA>Biochemistry,
**Neutral relationship**- It has been shown that cterRAP74 and the core domain of hTFIIB TFIIBc, residues 112 316 directly interact with the carboxyl terminal domain of hFCP1 cterFCP, residues 879 961, and these interactions may be responsible for the regulatory activities of TFIIF  [ **GTF2F1** ]  and TFIIB  [ **GTF2B** ]  on FCP1.  Ref: 12578358 Biochemistry< MedlineTA>Biochemistry,
- The interaction between RMP and TFIIF  [ **GTF2F1** ]  may be an additional passway for RMP to regulate the transcription, or alternatively TFIIF  [ **GTF2F1** ]  may cooperate with RPB5 and TFIIB  [ **GTF2B** ]  for the corepressor function of RMP.  Ref: 12737519 Cell Res, 2003
- Interestingly both RAP30 and RAP74  [ **GTF2F1** ]  interact with the same domain D5 of the C terminal RMP of 118 amino acid residuals which overlaps with its TFIIB  [ **GTF2B** ]  binding domain.  Ref: 12737519 Cell Res, 2003

**Non-interactive relationship** (e.g. studied together, co-existance, homology, etc.)

- Solution structure of the carboxyl terminal domain of RAP74  [ **GTF2F1** ]  and NMR characterization of the FCP1 binding sites of RAP74  [ **GTF2F1** ]  and human TFIIB  [ **GTF2B** ] .  Ref: 12578358 Biochemistry< MedlineTA>Biochemistry, 2003
- The measurements showed comparable amounts of RNA polymerase II, TFIIE, and TFIIF  [ **GTF2F1** ] , lower levels of TBP and TFIIB  [ **GTF2B** ] , and still lower levels of Mediator and TFIIH.  Ref: 11591727 J Biol Chem, 2001
- This model stipulates that wrapping of the promoter DNA around RNAPII and the general initiation factors TBP, TFIIB  [ **GTF2B** ] , TFIIE, TFIIF  [ **GTF2F1** ]  and TFIIH induces a torsional strain in the DNA double helix that facilitates strand separation and open complex formation.  Ref: 11509574 J Biol Chem, 2001
- Furthermore, we found that the recruitment of TFIIF  [ **GTF2F1** ]  and RNA polymerase II to a TFIID TFIIA TFIIB  [ **GTF2B** ]  promoter complex dictates the rate of preinitiation complex formation.  Ref: 11743717 J Mol Biol, 2001
- These results provide new information about the recognition of acidic rich activation domains involved in transcriptional regulation, and provide insights into how TFIIF  [ **GTF2F1** ]  and TFIIB  [ **GTF2B** ]  regulate the FCP1 phosphatase activity in vivo.  Ref: 12578358 Biochemistry< MedlineTA>Biochemistry, 2001
